# Integrating UAV-based multispectral imagery and machine learning to classify glyphosate-resistant palmer amaranth and assess herbicide program efficacy in soybean

**DOI:** 10.3389/fpls.2026.1913999

**Published:** 2026-10-26

**Authors:** Usman Mohammed, Prashant Jha, Vipan Kumar, Bhupesh Dhaka, Shahreen Mirza, Ivan Grijalva

**Affiliations:** 1 Research Associate, School of Plant, Environmental and Soil Sciences, Louisiana State University, Baton Rouge, LA, United States; 2 Professor; School of Plant, Environmental and Soil Sciences, Louisiana State University, Baton Rouge, LA, United States; 3 Associate Professor, Soil and Crop Sciences Section, Cornell University, Ithaca, NY, United States; 4 Graduate Research Assistant, School of Plant, Environmental and Soil Sciences, Louisiana State University, Baton Rouge, LA, United States; 5 Assistant Professor, Department of Entomology, Louisiana State University, Baton Rouge, LA, United States

**Keywords:** glyphosate resistance, herbicide program, integrated weed management, palmer amaranth, weed classification

## Abstract

Palmer amaranth is one of the most troublesome and economically important weeds in soybean production, with widespread occurrence of glyphosate-resistant (GR) populations. This study aimed to map and classify GR Palmer amaranth using UAV-based multispectral imagery and evaluate the effects of herbicide programs [preemergence (PRE) only, postemergence (POST) only, and PRE followed by (fb) POST] on Palmer amaranth coverage (early- and late-season) in soybean field in central Louisiana in 2025. A multispectral drone was used to map GR Palmer amaranth, and images were processed into orthomosaic using Pix4DFields and georeferenced in ArcGIS Pro. Weed classification was performed using pixel-based and object-based approaches implemented in ENVI and Orfeo Toolbox (OTB) within QGIS, employing Random Forest (RF), Multilayer Perceptron (MLP), and Support Vector Machine (SVM) algorithms. Both classification methods exhibited high performance, with RF achieving the highest overall accuracy (99%). UAV-based assessments provided a spatiotemporally efficient method for early-season Palmer amaranth mapping and late-season evaluation of residual weed activity following herbicide applications. UAV-derived Palmer amaranth coverage showed moderate positive correlation with the ground-truth weed density. Herbicide programs significantly reduced UAV-derived Palmer amaranth coverage during early-season (P = 0.0004) and late-season (P = 0.0223) assessments. Among the evaluated programs, PRE fb POST resulted in the greatest reduction in weed coverage (4.3%), compared with PRE only (9.2%) and POST-only (8.8%) programs. In conclusion, integrating UAV-based multispectral imagery with machine learning algorithms, particularly RF, offers a promising, automated approach for classification of GR Palmer amaranth and evaluating herbicide program effectiveness across different crop growth stages.

## 1 Introduction

Soybean is one of the world’s most important row crops, providing protein for food, feed, and industrial uses (Hamza et al., 2024; Hart, 2017; Monte et al., 2020). The United States accounts for approximately 28% of global soybean production (USDA-FAS, 2025). However, weed interference remains one of the major constraints to soybean productivity, with potential yield losses exceeding 50% in North America (Soltani et al., 2017). Several weed species, such as horseweed (*Erigeron canadensis L.)*, morningglories (*Ipomoea* spp.*)*, giant ragweed (*Ambrosia trifida L.)*, waterhemp (*Amaranthus tuberculatus L.)* and Palmer amaranth (*Amaranthus palmeri*) are of major concern, causing significant losses in soybean yield (McDonald et al., 2023; Norsworthy, 2003).

Palmer amaranth (*Amaranthus palmeri*) is one of the most troublesome and economically important weeds in U.S. soybean production because of its substantial impacts on crop yield and quality (Chahal et al., 2015; Heap, 2026). Bensch et al. (2003) reported soybean yield reductions of up to 79% at a Palmer amaranth density of 8 plants m^-2^. In Louisiana and neighboring southern states, Palmer amaranth is consistently ranked among the most troublesome weed species affecting soybean production (Stephenson et al., 2016).

Growers mostly rely on herbicides to manage Palmer amaranth in soybean. However, the extended emergence pattern, high growth rate, and prolific seed production combined with resistance to multiple herbicide sites of action make it difficult to control (Jha and Norsworthy, 2009; Kumar et al., 2019; Ward et al., 2013). To date, Palmer amaranth has evolved resistance to 10 different herbicide sites of action, with widespread occurrence of resistance to glyphosate in the Midsouth, first reported in 2010 in Louisiana (Heap, 2026).

Preemergence (PRE) herbicide program followed by postemergence (POST) can provide excellent Palmer amaranth control in soybean (Hoffner et al., 2012; Norsworthy et al., 2016). However, these programs are typically applied as broadcast treatments that do not account for the spatial variability of weed infestations within fields, highlighting the need for accurate weed mapping to support site-specific management (Huang et al., 2025; Sapkota et al., 2023).

Early detection and management of herbicide-resistant (HR) weed patches are essential for slowing the evolution and spread of HR populations (Beckie, 2006; Norsworthy et al., 2012). Site-specific weed management (SSWM) relies on accurate weed mapping to identify weed species and quantify their distribution, abundance, and spatial variability, enabling targeted herbicide applications (Kavhiza et al., 2020; San Martín et al., 2016). Accurate weed maps also improve the evaluation of herbicide efficacy and integrated weed management (IWM) practices while reducing selection pressure for herbicide resistance (Beckie, 2006).

Traditional ground-based weed assessment methods, such as visual rating and quadrants counts, are used to evaluate weed density and herbicide efficacy (Casanova et al., 2025; Duddu et al., 2019). However, these techniques are subjective, labor-intensive, and spatially discontinuous (Casanova et al., 2025; Duddu et al., 2019). The use of high-resolution approaches can improve weed mapping and support SSWM.

Previous studies have demonstrated that successful implementation of SSWM depends on accurate weed detection and the ability to capture spatial variation in weed densities across a field (Park and Krell, 2005; Park et al., 2007). Recent advancements in technology, particularly the integration of remote sensing, unmanned aerial vehicles (UAVs), and machine learning techniques have significantly enhanced weed detection by improving classification accuracy, enabling species level identification, and facilitating the generation of high-resolution weed distribution maps (Esposito et al., 2021; He et al., 2026; Lan et al., 2010; Wang et al., 2025). Recent studies have demonstrated the potential of UAV-based imaging combined with deep learning algorithms for weed detection and estimation of spatial distribution (Bretas et al., 2024; Ram et al., 2026). For example, Ram et al. (2026) developed a comprehensive UAV-based framework for Palmer amaranth detection and mapping, achieving a high mean average precision (mAP@0.5) of 0.97. Barnhart et al. (2022) used open-source software to detect Palmer amaranth from images collected using both handheld camera and a UAV, achieving a mAP of 77%. In a comparison of multiple convolutional neural network (CNN)-based object detection algorithms, Sharma et al. (2024) found that YOLOv9 provided the greatest accuracy in detecting Palmer amaranth seedlings, achieving a mAP @ 0.5 of 93.50% from handheld camera images. Similarly, Bretas et al. (2024) employed deep CNN to detect and map spiny pigweed using UAV imagery, achieving 94% accuracy. Although these studies have demonstrated the potential of UAV imagery and machine learning algorithms for accurate weed detection and classification, most have focused primarily on improving model performance metrics rather than evaluating the utility of UAV-derived weed maps for agronomic decision making (Marwah et al., 2026). Therefore, the relationship between UAV-derived weed estimates and conventional field assessments used for herbicide evaluation remains insufficiently understood. The objective of this study was to evaluate the potential of UAV-derived multispectral imagery and machine learning approaches for classifying glyphosate-resistant (GR) Palmer amaranth and assessing herbicide program efficacy in Louisiana soybean fields. Specifically, this study compared UAV-derived GR Palmer amaranth coverage estimates with conventional ground-based weed density assessments to determine their effectiveness for evaluating weed control outcomes among different herbicide programs.

## 2 Materials and methods

A field study was conducted in 2025 at the Louisiana State University Doyle Chambers Central Research Station (30.3625°N, 91.1628°W) Baton Rouge, LA. A glyphosate-, 2,4-D choline-, and glufosinate-resistant (Enlist^™^ E3) soybean was planted on April 4, 2025, at 55,059 seeds ha^-1^ into four row (97-cm spacing) plots measuring 7.6 m in length. The site had a natural uniform infestation of GR Palmer amaranth. The soil at the test site was Mhoon silty clay loam (fine-silty, mixed, nonacid, thermic Typic Flauaquent) with 1.9% OM and pH of 6.3.

The experiment was designed as a randomized complete block with four replications and one factor (herbicide program). Herbicide programs included preemergence (PRE) only, postemergence (POST) only, and PRE followed by POST (PRE fb POST). The PRE program comprised of *S*-metolachlor + fomesafen (Prefix^®^ herbicide, Syngenta Crop Protection, Greensboro, NC 27709) applied at 0.53 + 0.80 kg ai ha^-1^. POST treatment included glufosinate (Liberty^®^ 280 SL, BASF Corporation, Research Triangle Park, NC 27709) + 2,4-D choline (Enlist One^®^, Corteva Agriscience LLC, Indianapolis, IN 46268) applied at 0.66 + 1.06 kg ae ha^-1^ along with ammonium sulfate (Quest^®^, Helena Agri-Enterprises, LLC, Collierville, TN) at 0.5% v/v. PRE herbicides were applied immediately after soybean planting; POST herbicides were applied on June 5, 2025, corresponding to the V4-V5 stage of soybean. Plots assigned to the PRE- or POST-only treatments did not receive a water-only (mock) application at the alternate application timing, thus representing an incomplete factorial design. Palmer amaranth seedlings were 10 to 15 cm tall at the time of POST herbicide application. Herbicides were applied using a CO_2_-pressurized backpack sprayer calibrated to deliver 187 L ha^-1^ at 210 kPa using a four-nozzle spray boom equipped with TeeJet AIXR 11004 flat-fan nozzles (TeeJet, Spraying Systems Co., Wheaton, IL).

### 2.1 Data collection

Two drone flights were conducted over the study site using a DJI Mavic 3 Multispectral (SZ DJI Technology Co., Ltd., Shenzhen, China) equipped with multispectral camera. The camera captured four multispectral bands, including green (560 nm), red (650 nm), red-edge (730 nm), and near-infrared (NIR; 860 nm). The first flight was conducted on May 2, 2025 (28 DA-PRE and referred to as early-season flight), while the second was conducted on August 1, 2025 (56 DA-POST referred to as late-season flight). The early season flight was conducted before the POST application was made. Both flights followed a preset flight path at an altitude of 12 m and a speed of 1 m s^-^¹, with a forward overlap of 80% and a side overlap of 70%. Flights were carried out between 9:00 AM and 3:00 PM under minimal cloud cover and good visibility. A total of 1,300 images were collected for UAV-based weed classification using machine learning models. Prior to orthomosaic generation, radiometric calibration was performed using images of a calibrated reflectance panel collected before and after each flight. The calibration images were imported into Pix4DFields (version 2.9.7) to convert raw multispectral images into calibrated reflectance values. The calibrated images were then processed and stitched together to generate a single orthomosaic using Pix4DFields mapping software. The orthomosaic was georeferenced in ArcGIS Pro 3.4.0. using ground control points.

Ground-truth Palmer amaranth density was recorded from two 0.5 m^2^ permanent quadrats established at the center of each plot at a biweekly interval. Within each quadrat, Palmer amaranth seedlings were counted and then removed. Ground-truth data were used to correlate Palmer amaranth density with the UAV-derived percent coverage.

### 2.2 Weed classification

For GR Palmer amaranth classification, we employed two different classification methods: a) pixel and b) object-based classification. The image processing flowchart for both methods is shown in **Figure 1**.

**Figure 1:**
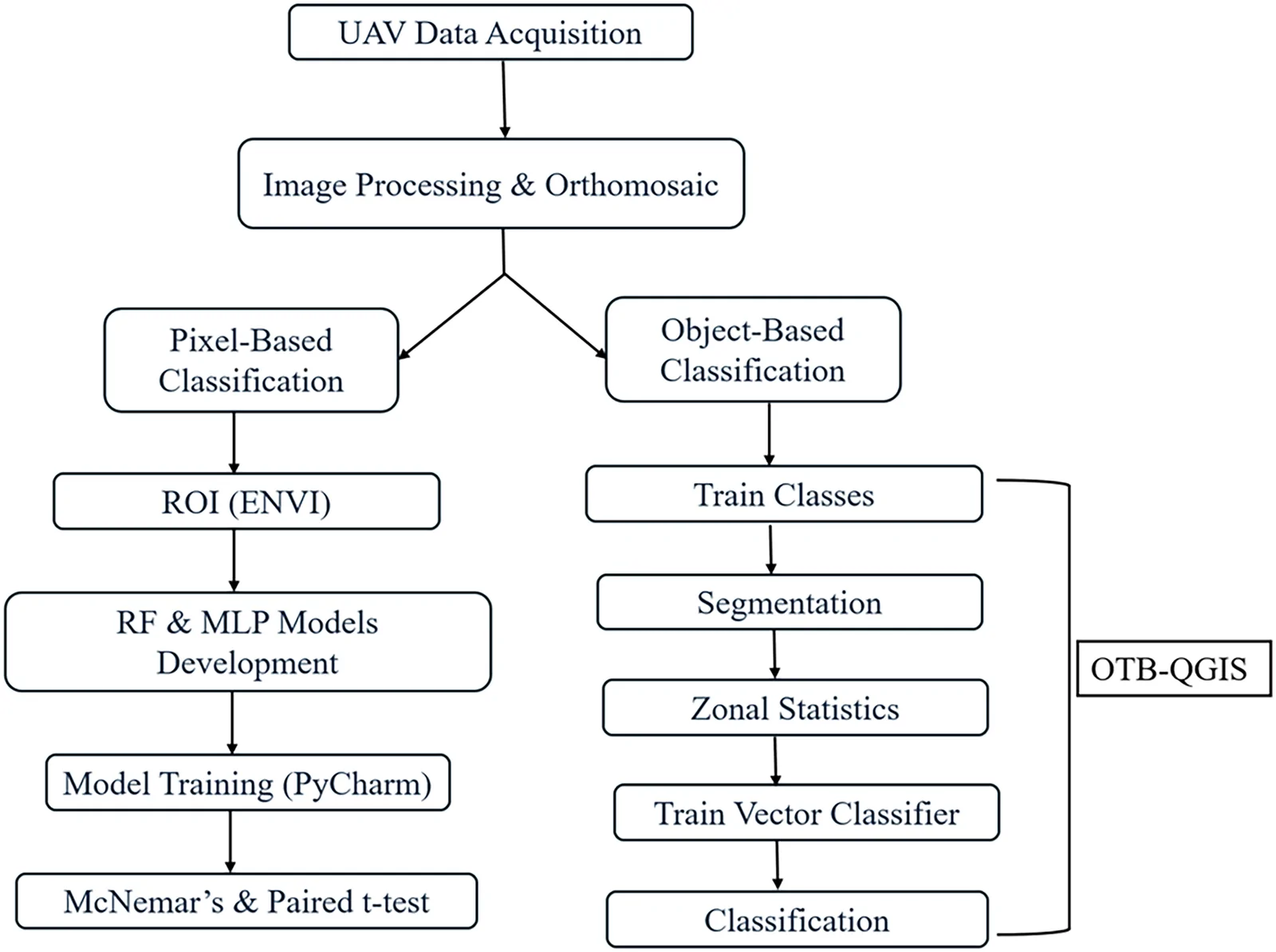
Image processing flowchart for pixel and object-based classification from data collection through model training and classification.

#### 2.2.1 Pixel-based classification

Pixel-based classification is a classification method that analyses each pixel independently and classifies them into predefined classes based on spectral similarities (Weih and Riggan, 2010).

Reference spectral information can be obtained by visually identifying representative regions or pixels corresponding to predefined classes, which can subsequently be used for pixel-level classification (Mohammed et al., 2026). In this study, regions of interest (ROIs) were visually identified for four classes: Palmer amaranth, soybean, soil, and other weed species. ROIs were randomly selected throughout the orthomosaic (1.03 cm spatial resolution) to capture spatial and spectral variability associated with different field conditions and vegetation characteristics. The final dataset consisted of 175 ROIs, including 42% Palmer amaranth, 28% soybean, 15% soil, and 15% other weed species. Although the number of manually delineated ROIs was limited, each ROI contained multiple pixels with multispectral information, providing multiple spectral observations for pixel-based classification. The unequal distribution among classes reflects the availability of representative areas within the study field and should be considered when interpreting model performance.

Random Forest (RF) and Multi-Layer Perceptron (MLP) classifiers were used for pixel-based classification. RF is an ensemble of unpruned classification created by using bootstrap samples of the training data and random feature selection in tree induction (Svetnik et al., 2003). RF can handle high dimensional data and has the ability to ignore irrelevant descriptors (Svetnik et al., 2003). RF has previously been applied for weed detection and mapping using UAV imagery (Sheffield et al., 2022; Singh and Singh, 2022). de Castro et al. (2018) employed RF for early weed mapping between and within crop rows using UAV imagery. While MLP is an adaptive layered neural network used for classification (German, 1995). It consists of multiple layers of interconnected neurons that learn through backpropagation algorithms to automatically classify data (German, 1995; Pacífico et al., 2018). MLP typically includes input, hidden, and output layer, with the number of hidden layer neurons being adjustable to optimize performance (Pacífico et al., 2018). MLP has been used for seed, weed, and plant disease classification (Abdullah and Quteishat, 2015; Garg et al., 2025; Tamouridou et al., 2017).

Both RF and MLP models were implemented in Python using the scikit-learn library (Pedregosa et al., 2011). For RF classification, the scikit-learn RandomForestClassifier was implemented using default hyperparameter settings with a fixed random seed (random_state = 42) to ensure reproducibility. The RF model consisted of 100 decision trees (n_estimators = 100), no predefined maximum tree depth (max_depth = None), and the square root of the available features was considered at each split (max_features = "sqrt").

For MLP classification, input features were standardized using the StandardScaler method before model training. The MLP model was implemented using the scikit-learn MLPClassifier with one hidden layer containing 100 neurons (hidden_layer_sizes = (100,)). The model used the ReLU activation function, Adam optimization algorithm, an initial learning rate of 0.001, and a maximum of 1000 iterations (max_iter = 1000). A fixed random seed (random_state = 42) was applied to improve reproducibility.

Both RF and MLP models were evaluated using stratified 5-fold cross-validation (StratifiedKFold; n_splits = 5), with random shuffling and preservation of class proportions among folds. During each iteration, four folds were used for model training and the remaining fold was used for validation. The reported classification metrics were calculated from predictions aggregated across all five validation folds.

#### 2.2.2 Object-based classification

Object-based classification is an advanced classification method that classifies images based on object properties or regions rather than individual pixels (Blaschke, 2003). It incorporates information such as shape, texture, and contextual features of image objects (Blaschke, 2003; Marpu et al., 2008). In this study, the object-based classification was performed in different steps using Orfeo Toolbox (OTB) plugin integrated within QGIS. The first step involved creating training datasets that served as ground truth for classification. The training datasets were collected from the orthomosaic by means of point vector layers and the orthomosaic was segmented using Mean Shift algorithm (De Luca et al., 2019). The segmentation algorithm grouped pixels based on spectral similarity and spatial proximity, producing relevant homogenous regions. The output of the segmentation process was a segmented vector layer with different polygons, each representing a potential class.

For each segment, zonal statistics (mean, median, and standard deviation) were calculated for all relevant spectral bands, and pixel data were converted into vector-based attributes and a feature set for classification. The training classes were then joined to the segmented polygons based on their spatial location, and only labelled segments were used for the training. The labelled image objects were classified using a Support Vector Machine (SVM) classifier with the *libsvm* model. Thus, Mean Shift was used for image segmentation, while SVM was used as the classifier for the resulting objects. The final maps were visualized using class-specific symbols (**Figure 2**).

**Figure 2:**
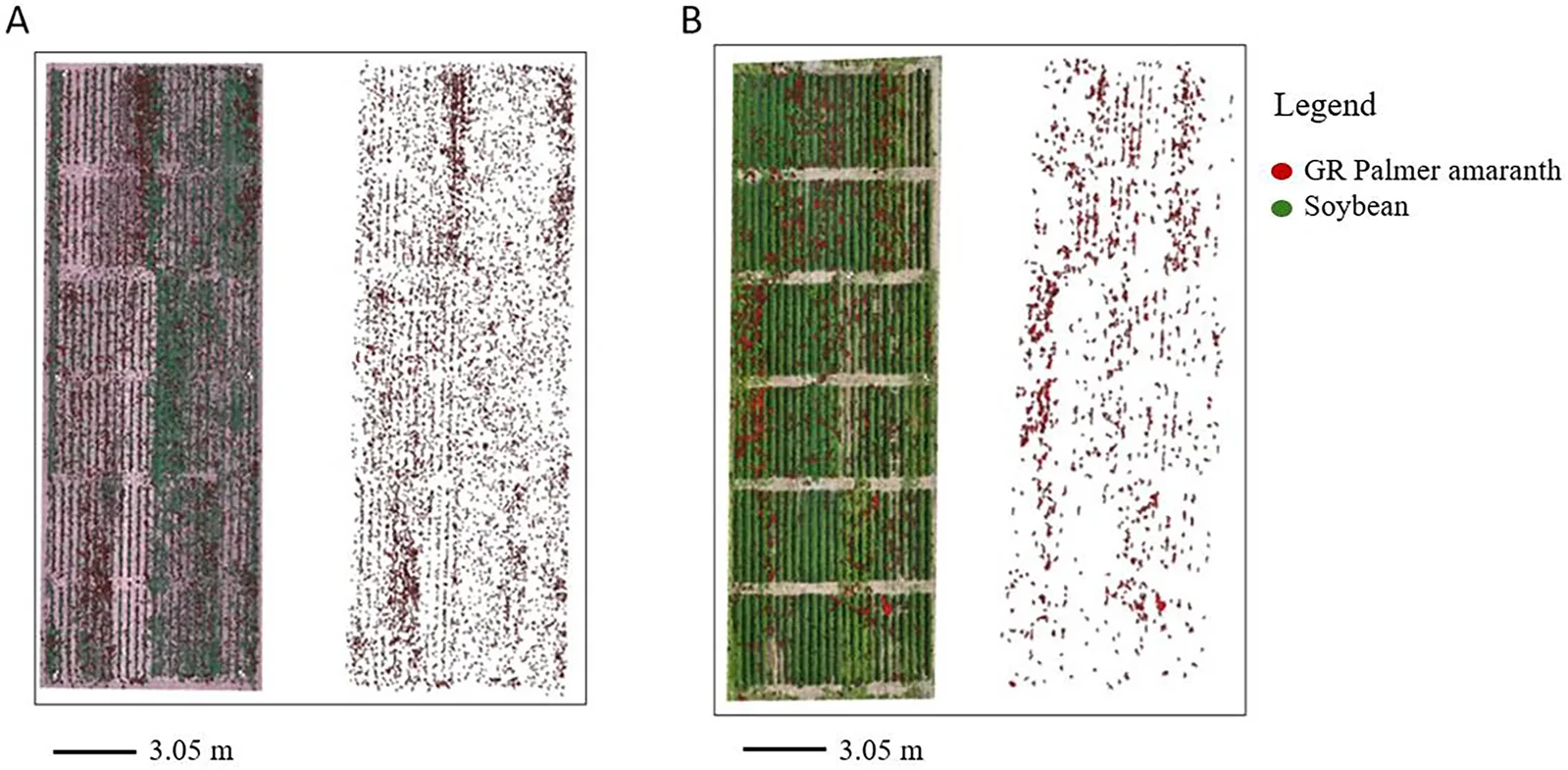
Classification maps showing distribution of glyphosate-resistant (GR) Palmer amaranth (red area) and soybean (green area) during A) early-season flight and B) late-season flight. For each panel, the left image shows the classified map of both GR Palmer amaranth and soybean, while the right image shows the masked-out map displaying the distribution of GR Palmer amaranth.

### 2.3 Statistical analysis

All statistical analyses were conducted in PyCharm 2025.1.2.

The effect of herbicide program on UAV coverage was analyzed separately for early- and late-season flights. Statistical test was selected based on assumption framework (Arredondo, 2026). Normality and homogeneity of variance were assessed using Shapiro-Wilk and Levene’s tests, respectively. Within each flight, the effect of herbicide program was analyzed using one-way ANOVA, followed by Tukey’s HSD post-hoc test when treatment effects were detected. Temporal changes between flights were assessed within each herbicide program using paired t-test (P<0.05) or Wilcoxon signed-ranked tests when assumptions were not met.

For the comparison of pixel-based classification algorithms, the performance of RF and MLP classifiers were compared using McNemar’s test (Gao et al., 2018). McNemar’s test is a non-parametric statistical test used to assess the difference in classification accuracy between two or more models (Roggo et al., 2003). The test was conducted separately for each flight (early and late season) using a threshold of P<0.05. For the comparison of object-based classification algorithms, confusion matrix was generated using the OTB plugin in QGIS. A confusion matrix is a two-dimensional contingency table used to visualize model performance by showing correct and incorrect predictions (Heydarian et al., 2022). Using the confusion matrix, overall accuracy, per-class precision, recall, and F1 score were calculated using Equations 1–4 (Sathyanarayanan, 2024; Sujon et al., 2025; Tharwat, 2021).

(1) $$\text{Overall Accuracy}=\frac{TP\;+TN}{TP\;+\;TN+FP+FN}$$

where TP, TN, FP, and FN represent true positive, true negative, false positive, and false negative, respectively.

Precision measures the proportion of positive predictions that are correct:

(2) $$\text{Precision}=\frac{TP\;}{TP\;+\;FP}$$

Recall measures the proportion of actual positive cases that are correctly identified:

(3) $$\text{Recall}=\frac{TP\;}{TP\;+\;FN}$$

F1-score is the harmonic mean of precision and recall, providing a balance between them:

(4) $$\text{F}1\text{-score}=\frac{2\;\left(Precision\;\times \;Recall\right)\;}{Precision\;+\;Recall}$$

The relationship between UAV-derived Palmer amaranth coverage (%) and ground-truth Palmer amaranth density (plants m^-2^) was evaluated using Spearman's rank correlation analysis. A separate negative binomial generalized linear model (NBGLM) was fitted to account for overdispersion in Palmer amaranth count data (Payne et al., 2017; Wiles et al., 1992).

## 3 Results

### 3.1 Effect of herbicide programs on palmer amaranth UAV coverage

The effect of herbicide programs, including PRE only, POST only, and PRE fb POST, was significant on Palmer amaranth UAV coverage for both early- and late-season flights (**Figure 3**). During the early-season flight, Palmer amaranth coverage differed significantly among treatments (P = 0.0004). The PRE only and PRE fb POST programs were more effective in controlling Palmer amaranth, resulting in the lowest UAV-derived coverage values of 9.5% and 9.9%, respectively, compared with 17.5% coverage for the POST-only program. This difference was attributed to the timing of herbicide application, as the POST treatment was applied after the early-season flight.

**Figure 3:**
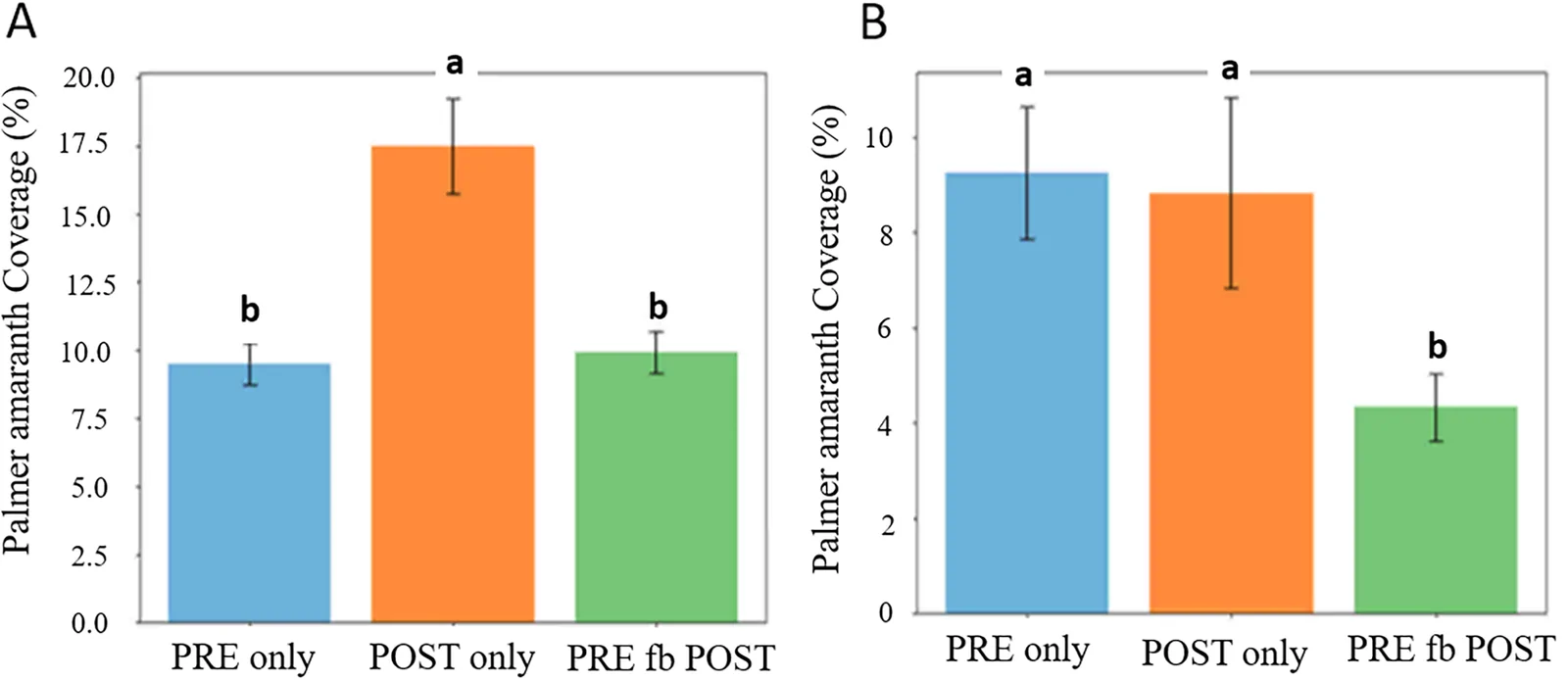
Effect of herbicide program on GR Palmer amaranth UAV-derived coverage during **(A)** early-season flight and **(B)** late-season flight. Bars represent mean ± standard error (SE). Means followed by the same letter are not significantly different according to Tukey’s HSD test (P<0.05).

During the late-season flight, which was conducted after the POST herbicide application, a significant reduction in UAV-derived Palmer amaranth coverage was observed among herbicide programs (P = 0.0223). The PRE fb POST program provided the greatest reduction in Palmer amaranth coverage, with 4.3% coverage compared with 9.2% and 8.8% coverage for the PRE-only and POST-only programs, respectively. These results demonstrate that integrated PRE fb POST herbicide programs were more effective in reducing Palmer amaranth coverage throughout the growing season.

### 3.2 Weed classification

The classification accuracies are presented in **Table 1**. For pixel-based classification, both RF and MLP exhibited high classification performance based on 5-fold cross-validation results. During the early-season flight, MLP achieved slightly higher accuracy than RF; however, no significant difference in accuracy was detected between the two algorithms (P = 0.7905). In contrast, during the late-season flight, RF achieved higher classification accuracy than MLP, with a significant difference between algorithms (P = 0.0078). The highest overall classification accuracy was achieved using RF during the late-season flight (99%).

**Table 1:** Pixel and object-based classification accuracy for early- and late-season classification of Palmer amaranth in soybean. Pixel-based classification accuracies (RF and MLP) were calculated using stratified 5-fold cross-validation with 175 samples. Object-based classification accuracy was calculated using 188 validation objects.

Model	Validation samples (n)	Accuracy (%)	
		Early season	Late season
Pixel-Based: Random Forest (RF)	175	90	99
Pixel-Based: Multi-Layer Perceptron (MLP)	175	94	93
Object-Based: Support Vector Machine (SVM)	188	75	84

Model performance varied with flight timing. During the early-season flight, MLP showed a slightly higher classification performance than RF, whereas RF achieved superior classification performance during the late-season flight (**Tables 2**, **3**). Both models provided reliable discrimination between Palmer amaranth and soybean during the late-season flight, while class-specific performance varied during the early season. Object-based classification showed lower performance than the pixel-based classification approaches (RF and MLP), particularly for Palmer amaranth detection, although soybean classification was comparatively stronger across both flight timings (**Tables 2**, **3**).

**Table 2:** Per-class precision, recall, and F1-score for early-season classification of glyphosate-resistant (GR) Palmer amaranth in soybean.

Model	Palmer amaranth			Soybean		
	Precision (%)	Recall (%)	F1-score (%)	Precision (%)	Recall (%)	F1-score (%)
Pixel-Based: Random Forest (RF)	92	91	91	86	88	87
Pixel-Based: Multi-Layer Perceptron (MLP)	95	96	96	94	92	93
Object-Based: Support Vector Machine (SVM)	71	56	62	82	75	78

**Table 3:** Per-class precision, recall, and F1- score for late-season classification of glyphosate-resistant (GR) Palmer amaranth in soybean.

Model	Palmer amaranth			Soybean		
	Precision (%)	Recall (%)	F1-score (%)	Precision (%)	Recall (%)	F1-score (%)
Pixel-Based: Random Forest (RF)	99	100	99	100	98	99
Pixel-Based: Multi-Layer Perceptron (MLP)	95	93	94	90	92	91
Object-Based: Support Vector Machine (SVM)	78	53	63	78	93	85

### 3.3 Relationship between UAV coverage and ground-truth GR palmer amaranth density after herbicide application

The relationship between UAV-derived Palmer amaranth coverage (%) and ground-truth Palmer amaranth density (plants m^-2^) was positive during both the early- and late-season assessments (**Figure 4**). Spearman's rank correlation analysis showed significant moderate positive associations between UAV-derived coverage and ground-truth Palmer amaranth density during the early-season assessment (ρ = 0.54, P = 0.0006) and the late-season assessment (ρ = 0.53, P = 0.0008). In both assessments, plots with greater UAV-derived Palmer amaranth coverage generally exhibited higher ground-truth Palmer amaranth densities.

**Figure 4:**
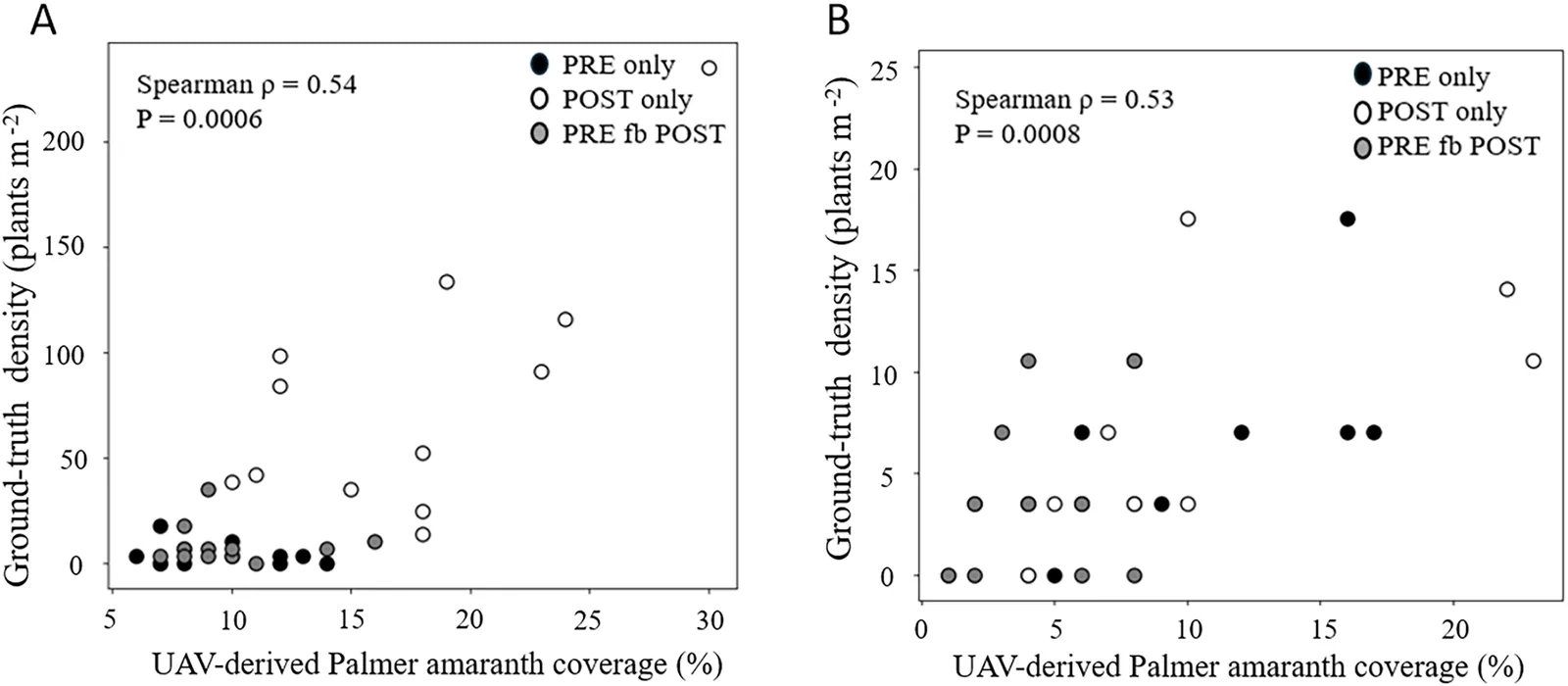
Correlation between UAV-derived Palmer amaranth coverage (%) and ground-truth Palmer amaranth density (plants m^-2^) during **(A)** early-season and **(B)** late-season assessments. Spearman's rank correlation coefficients (ρ) and corresponding *P*-values are shown. Marker styles represent herbicide treatments (PRE only, POST only, and PRE fb POST).

A separate negative binomial generalized linear model (NBGLM) was fitted to account for overdispersion in Palmer amaranth count data. Model performance differed between assessment periods, with pseudo-R^2^ values of 0.78 for the early-season assessment and 0.16 for the late-season assessment.

## 4 Discussion

### 4.1 Effect of herbicide programs on palmer amaranth UAV coverage

Herbicide programs were effective in managing Palmer amaranth, with PRE fb POST program providing the highest reduction in UAV-derived Palmer amaranth coverage by the late season assessment. The results are consistent with previous findings that PRE fb POST herbicide programs are more effective than a single PRE or POST program for managing Palmer amaranth, which continues to emerge in multiple flushes from mid-April through August in soybean production systems in the Southern U.S (Jha and Norsworthy, 2009). Sarangi and Jhala (2019) reported Palmer amaranth control of 66% with a PRE-only program compared with 86% control from a PRE fb POST program in soybean. Similarly, Kaur et al. (2024) reported that Palmer amaranth control did not exceed 78% with PRE or POST-only programs compared with 94% control with a PRE fb POST program in soybean.

During the late-season flight, the POST-only program had slightly lower UAV-derived percent coverage than the PRE-only program, indicating the importance of extended residual activity from soil-applied PRE herbicides with multiple sites of action for managing glyphosate-resistant Palmer amaranth in soybean (Carvalho-Moore et al., 2025; Norsworthy et al., 2012). When comparing herbicide programs across flight timings (**Figure 5**), POST-only (P = 0.0081) and PRE fb POST (P = 0.0007) programs showed a significant reduction in Palmer amaranth UAV-derived percent coverage during the late-season flight compared with the early-season flight. However, no significant reduction in coverage between early- and late-season flights was observed for the PRE-only program.

**Figure 5:**
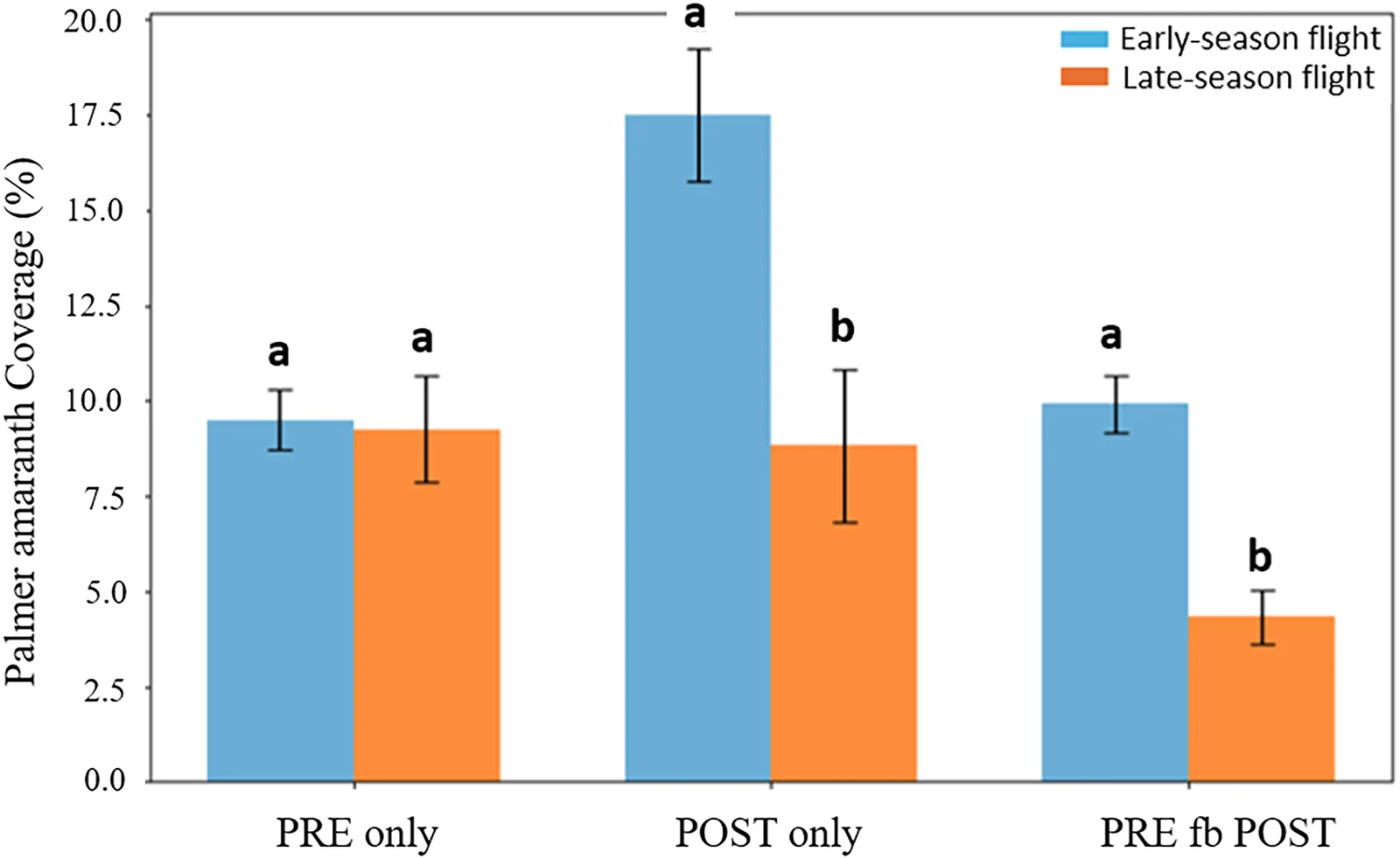
GR Palmer amaranth coverage in early-season vs. late-season flight under each herbicide program [preemergence (PRE) only, postemergence (POST) only, and PRE followed by (fb) POST] in soybean. Bars represent mean ± standard error (SE). Different letters indicate significant differences between flights within a herbicide program (P<0.05).

These results further demonstrate the extended residual activity of fomesafen and S-metolachlor on Palmer amaranth until 56 DA-POST. Chudzik et al. (2026) reported greater soil retention of fomesafen in fine-textured silt loam soils, resulting in improved weed control later in the season. Similarly, Hoffner et al. (2012) reported that fomesafen plus S-metolachlor provided up to 80% control of Palmer amaranth 6 weeks after soybean planting. Overall, UAV-derived coverage provided an effective approach for detecting differences among herbicide programs and evaluating treatment performance throughout the growing season.

### 4.2 Weed classification

The overall classification accuracy of RF and MLP was high for both early- and late-season flights. The highest overall classification accuracy (99%) was achieved using RF during the late-season flight. The results of pixel-based classification were consistent with previous studies using UAV-based approaches for weed detection (Mohammed et al., 2026; Tamouridou et al., 2017). Sanders et al. (2021) reported 69 to 90% accuracy for Palmer amaranth classification, while Tamouridou et al. (2017) achieved 99.9% accuracy using multispectral imagery and Multi-Layer Perceptron with Automatic Relevance Determination (MLP-ARD). Additionally, Bento et al. (2023) reported 99.24% accuracy using RF for weed detection in coffee fields.

RF is effective in handling small sample sizes, complex data structures, and nonlinear relationships, which contributes to its high classification accuracy (Cutler et al., 2007). In contrast, MLP models generally require larger datasets and improve as training data increases because of their ability to learn complex patterns (Omurlu et al., 2020). Although the number of manually delineated ROIs was relatively limited (175 ROIs), each ROI contained multiple pixels with multispectral information, providing numerous spectral observations for pixel-based classification. The use of 5-fold cross-validation, where each ROI was used for validation during one iteration, reduced the likelihood of overestimating model performance (Kriegeskorte, 2015). However, potential overfitting cannot be completely excluded, particularly given the high accuracy achieved by RF during the late-season flight. Future studies should validate these models using larger datasets across diverse fields, weed densities, and environmental conditions to improve model generalizability.

For individual classes, both models achieved high precision, recall, and F1-scores, demonstrating their robustness (**Table 2** and **3**). During the early-season flight, both models showed slightly higher precision, recall, and F1-scores for Palmer amaranth compared with soybean. During the late-season flight, this pattern was less distinct for RF, as both species demonstrated similar performance across all three metrics. Palmer amaranth showed slightly lower precision but higher recall than soybean, indicating a trade-off between predictive confidence and detection ability.

RF performance improved during the late-season flight when Palmer amaranth and soybean had greater biomass and canopy development. This finding agrees with Shang et al. (2011), who reported the highest classification accuracy during mid- to late-season measurements when crops had greater biomass and canopy coverage. Although RF and MLP performance vary depending on dataset characteristics (Bhatt and Maclean, 2023; Sumsion et al., 2019), higher prediction accuracy is generally achieved later in the season when vegetation structure is more developed.

When comparing pixel- and object-based classification methods, previous studies have reported that object-based classification can outperform pixel-based approaches for weed and vegetation mapping because segmentation incorporates spatial context, shape, and texture information (Pérez-Ortiz et al., 2015; Yan et al., 2006; Yu et al., 2006). However, in the current study, pixel-based classification achieved higher accuracy than object-based classification. This difference may be attributed to the characteristics of the target weeds, sampling design, image spatial resolution, and segmentation performance (Ubben et al., 2024; Yuba et al., 2021). During the early season flight, Palmer amaranth seedlings were small with limited canopy development, making object-based segmentation more challenging. Individual seedlings occupied few pixels and were often spectrally mixed with soil, crop residue, and surrounding vegetation, which can lead to inaccurate object boundaries and loss of subtle spectral differences (Ronay et al., 2022). In contrast, pixel-based classification retained the original spatial resolution and enabled RF and MLP to capture fine-scale spectral variation among weed, crop, soil, and residue classes. Although both approaches benefited from increased biomass and improved spectral separation later in the season, the higher performance of pixel-based methods suggests that preserving pixel-level spectral information was more important than object-level spatial characteristics under the conditions evaluated.

### 4.3 Relationship between UAV coverage and ground-truth GR palmer amaranth density after herbicide application

The positive relationship observed between ground-truth Palmer amaranth density and UAV-derived Palmer amaranth coverage indicates that UAV imagery effectively captured spatial patterns of Palmer amaranth across treatment plots. Moderate correlations were observed during both the early-season (ρ = 0.54) and late-season (ρ = 0.53) assessments, demonstrating that greater UAV-derived Palmer amaranth coverage was generally associated with higher field-measured Palmer amaranth densities. However, the moderate correlation coefficients also indicate that UAV-derived coverage did not fully correspond with variations in ground-truth density across all plots.

Several factors may have contributed to the observed variability between UAV-derived coverage estimates and ground-truth Palmer amaranth density. Misclassification of non-target weed species, overlapping weed canopies, and increasing soybean canopy interference later in the growing season may have affected the accuracy of UAV-derived coverage estimates. Variability between UAV-derived coverage estimates and ground-truth Palmer amaranth density likely resulted from misclassification of non-target species, overlapping canopies, and differences in spatial scale between UAV imagery and field sampling. The lower late-season model performance (pseudo-R² = 0.16) suggests a limitation caused by reduced spectral separation between senescing soybean and green Palmer amaranth. As crop senescence progressed, mixed spectral responses increased classification uncertainty. Thus, UAV imagery may be most effective for detecting Palmer amaranth patterns during earlier growth stages before canopy closure and senescence reduce spectral distinction. For example, Randelović et al. (2020) reported weaker correlations between UAV-derived and ground-based weed measurements in plots with higher soybean plant density. In addition, differences in spatial scale between ground-truth sampling locations and UAV-derived coverage estimates may have contributed to the reduced prediction accuracy (Canicattì et al., 2026). Despite these limitations, UAV-derived Palmer amaranth coverage provided a useful indicator of Palmer amaranth abundance and demonstrated potential for supporting rapid weed assessment and site-specific management decisions.

Future research should further investigate the relationship between UAV-derived Palmer amaranth coverage and ground-truth Palmer amaranth density across diverse crop canopies, weed infestation levels, and herbicide management programs. Additional efforts should focus on reducing interference from non-target weed species and evaluating higher-resolution imagery and advanced classification approaches to improve density estimation accuracy.

## Data Availability

The raw data supporting the conclusions of this article will be made available by the authors, without undue reservation.
